# Insights into an innovative point of care ultrasound curriculum for Ontario primary maternity care providers

**DOI:** 10.12688/mep.19361.1

**Published:** 2022-10-26

**Authors:** Bronte K. Johnston, Elizabeth Darling, Anne Malott, Susan Kras, Carol Bernacci, Laura Thomas, Beth Murray-Davis

**Affiliations:** 1 McMaster Midwifery Research Centre, McMaster University, Hamilton, ON, L8N 3Z5, Canada; 2 School of Population and Public Health, University of British Columbia, Vancouver, BC, V6T 1Z8, Canada; 3 Mohawk College and McMaster Medical Radiation Sciences Program, McMaster University Mohawk College, Hamilton, Ontario, L8S 1C7, Canada

**Keywords:** point of care ultrasound training, midwifery continuing education, primary pregnancy care

## Abstract

Point of care ultrasound (POCUS) has increasingly been used by midwives worldwide. In 2018, the scope of midwifery care in Ontario was expanded to include POCUS to allow practitioners to provide more comprehensive care. In response to the scope expansion, a new continuing POCUS education course was created in collaboration with faculty and clinicians from obstetrics, midwifery, and medical radiation sciences. The continuing education sonography course focused on fostering the knowledge, skills and judgment Ontario midwives required to safely perform these new POCUS skills. The course included online modules, a two-day hands-on bootcamp workshop, and a clinical practicum under the supervision of a sonographer to confirm competency across the three trimesters of pregnancy. The first cohort of 17 learners completed the course in Fall 2019, the new curriculum was well received by learners for its many benefits into learning and applying bedside sonography to clinical care. This paper outlines our process for POCUS curriculum development and implementation in pregnancy care. This POCUS continuing education course should continue to be offered in the future to give more practitioners the ability to perform point of care pregnancy scans.

## Introduction

Point of care ultrasound (POCUS) allows healthcare providers to perform bedside ultrasound scans for their patients and is often used in conjunction with a physical exam to aid clinical decision making ( Canadian Association of Radiologists Position POCUS Statement). Globally, midwives provide primary maternity care during pregnancy, birth and up to six-weeks postpartum. Many use POCUS to aid in clinical assessments such as pregnancy dating and confirming fetal presentation ( Bentley *et al*., 2015; Edvardsson *et al*., 2016; Kimberly *et al*., 2010; Vinayak *et al*., 2017; Vinayak & Brownie, 2018). In 2018, the College of Midwives of Ontario (CMO) added sonography to the scope of practice ( Scope of Practice Changes -Ultrasound). Therefore, there was a need to design a sonography course to support qualified midwives to gain the necessary knowledge, skills, and clinical judgment in these technologies.

## Ethics approval

Research regarding the McMaster-Mohawk POCUS course received ethics approval from the Hamilton Integrated Research Ethics Board project number: 7525.

## Results

From December 2018-November 2019, McMaster’s Midwifery Education Program partnered with the McMaster and Mohawk College Medical Radiation Sciences Program, to design and implement a continuing education course for primary maternity care providers, the first of its kind in Ontario. The core curriculum team of five midwifery and sonography faculty members met monthly for over a year to develop the course and the curriculum. Two Maternal Fetal Medicine specialists also met with the core team as needed to assist in defining course learning objectives for the course as well as to review and vet module content. 

The course objectives were informed by a needs assessment examining intended uses of ultrasound among Ontario midwives as the curriculum was guided by the obstetrical competency requirements from Sonography Canada Sonography Canada Guidelines and Member Policies 2019 ( Ling, 2019). The format for the course was based on two existing continuing education courses – emergency skills in obstetrics and a point of care ultrasound in the emergency room. Overall, the course encompassed five online modules, a two-day hands-on workshop, and clinical practicum guided by a placement logbook in which learners’ competencies are reviewed by a sonography preceptor.

Online module topics included: sonography ethics and basics, the physics of ultrasound, and sonographic protocols throughout pregnancy. The modular curriculum was primarily created by the sonography faculty, who drew on their obstetrics content and learning objectives from their existing medical radiation sciences program. Content related to how sonography competencies apply to the midwifery scope of practice, including discussions of the ethics and limitations of ultrasound was created by the midwifery faculty.

The hands-on workshop allowed learners to practice the clinical skills introduced in the modules with computer simulations and pregnant volunteers. Over 40 pregnant volunteers across all stages of pregnancy were recruited through midwifery clinics in Hamilton Ontario via social media and clinic flyers to provide simulated learning that would closely represent their clients. After successful completion of the workshop, learners organized a community practicum to further develop their skills under the guidance of a preceptor.

Learner knowledge, skills, and attitudes were continuously assessed across the 3 modalities of instruction. Participants were required to complete modules and attain a minimum of 80% on formative quizzes before attending the workshop. Ultrasound skills were evaluated in the workshop through a series of simulated scans an objective structural clinical exam (OSCE). These respective assessments were developed by the sonography faculty based on their existing other medical radiation sciences OSCE content. Learners were required to pass the OSCE to progress to the practicum and remediation was available if required. In the practicum, learners maintained a logbook of completed first, second and third trimester scans, all of which were evaluated by a sonography preceptor. Logbook criteria were developed and implemented by sonography faculty based on midwifery learning objectives, and experience needed to confirm proficiency. Successful completion of all course materials and assessments entitled learners a certificate of course completion so they were able to begin clinically applying POCUS.

Seventeen midwives enrolled and completed the POCUS course. Learners’ course evaluation surveys indicated that they appreciated the diversity of topics and educational methods used. The midwives shared how the course gave them a well-rounded understanding of the knowledge and clinical skills required to incorporate POCUS throughout antenatal care. Participants indicated they would use bedside scanning most frequently to assess viability and confirmation of fetal presentation. Certain aspects of the course were challenging for learners; for example, the physics module was cited as being difficult due to the depth and volume of content. It was also noted that the course and the gaining of new ultrasound skills took more time than anticipated. Finally, due to challenges with the coronavirus disease 2019 (COVID-19) pandemic, with limited availability of clinical sites to take on additional learners, many struggled to complete the clinical practicum.

## Discussion

The interprofessional collaboration during the curriculum development and roll out of this course ensured that the content was strongly rooted in pregnancy-specific sonography competencies. The course was a feasible and effective approach for promoting knowledge, skills, and judgment for qualified midwives to add POCUS to their clinical scope of practice. Strengths of this curriculum included the use of various teaching and learning modalities that encompassed didactic and clinical skills teachings and a clinical practicum guided by competency-based assessments.

Reflecting on the first cohort, the inclusion of theoretical knowledge and practical skills was very important to ensure that providers could successfully and appropriately perform a POCUS scan. Although some commented on the material being difficult, this content needs to be continued, potentially over a longer time-period as sonography is a complex, operator-dependent modality. The issues midwives experienced in coordinating their clinical practicum, is a continued theme across clinical education programs. Organizing placements is often difficult because the increased number of learners and limited resources available in clinical settings ( Larue *et al*., 2015). Potentially in this POCUS course, instructors could provide more time on the ultrasound simulator with pregnant volunteers in the workshop to reduce the number of scans required in the placement or sonography preceptors providing skills instruction in a midwifery clinic. Learners’ positive reception to the course re-iterates midwives’ interest in using POCUS to aid their clinical decisions.

## Conclusions and future directions

The education offered in the McMaster-Mohawk POCUS course was valuable and well received by midwives. Based on the first cohort, it is intended that the course will run annually.

In sharing our experiences with designing and implementing this POCUS course, we hope to provide guidance for curricula developers to help make POCUS more widely available in clinical care.

## Data Availability

No data are associated with this article.
