# Insights into an innovative point of care ultrasound curriculum for Ontario primary maternity care providers

**DOI:** 10.12688/mep.19361.3

**Published:** 2025-02-24

**Authors:** Bronte K. Johnston, Elizabeth Darling, Anne Malott, Susan Kras, Carol Bernacci, Laura Thomas, Beth Murray-Davis

**Affiliations:** 1 McMaster Midwifery Research Centre, McMaster University, Hamilton, ON, L8N 3Z5, Canada; 2 School of Population and Public Health, University of British Columbia, Vancouver, BC, V6T 1Z8, Canada; 3 Mohawk College and McMaster Medical Radiation Sciences Program, McMaster University Mohawk College, Hamilton, Ontario, L8S 1C7, Canada

**Keywords:** point of care ultrasound training, midwifery continuing education, primary pregnancy care

## Abstract

Point of care ultrasound (POCUS) has increasingly been used by midwives worldwide. In 2018, the scope of midwifery care in Ontario was expanded to include POCUS to allow practitioners to provide more comprehensive care. In response to the scope expansion, a new continuing POCUS education course was created in collaboration with faculty and clinicians from obstetrics, midwifery, and medical radiation sciences. The continuing education sonography course focused on fostering the knowledge, skills and judgment Ontario midwives required to safely perform these new POCUS skills. The course included online modules, a two-day hands-on bootcamp workshop, and a clinical practicum under the supervision of a sonographer to confirm competency across the three trimesters of pregnancy. This paper outlines the process for POCUS curriculum development and implementation in pregnancy care following course completion. The first cohort of 17 learners completed the course in 2019. The new curriculum was well received by learners for learning and applying bedside sonography in clinical care. The indications to use POCUS most frequently reported by learners after course completion included assessment of pregnancy viability and fetal presentation. Challenges identified by participants with the course included learning new content such as physics and struggling to complete the clinical practicum due to the coronavirus pandemic. The success of this course is indicated by the completion of the objective structured clinical exams for all learners and meeting the competencies for beginning their practicum. The POCUS continuing education course plays an important role in providing the knowledge, skills and ability to perform point of care pregnancy scans among midwives.

## Introduction

Point of care ultrasound (POCUS) allows healthcare providers to perform bedside ultrasound scans for their patients and is often used in conjunction with a physical exam to aid clinical decision making ( Canadian Association of Radiologists Position POCUS Statement). Globally, midwives provide primary maternity care during pregnancy, birth and up to six-weeks postpartum. Many use POCUS to aid in clinical assessments such as pregnancy dating and confirming fetal presentation ( Bentley *et al*., 2015; Edvardsson *et al*., 2016; Kimberly *et al*., 2010; Vinayak & Brownie, 2018; Vinayak *et al*., 2017). In 2018, the College of Midwives of Ontario (CMO) added sonography to the scope of practice. In order to apply POCUS in clinical practice, midwives are required to demonstrate the required sonography knowledge, clinical judgement, and skills ( Scope of Practice Changes -Ultrasound). However, despite this identified need for clinical application, there were no education training programs to teach midwives this skill set. To address the requirements outlined CMO for midwives to use bedside scanning, there was a need to design a sonography course to support qualified midwives to gain the necessary knowledge, skills, and clinical judgment in these technologies so POCUS can be included in their clinical work ( Ling, 2019).

## Ethics approval

Research regarding the McMaster-Mohawk POCUS course received ethics approval from the Hamilton Integrated Research Ethics Board project number: 7525.

## Results

From December 2018-November 2019, McMaster’s Midwifery Education Program partnered with the Mohawk College Medical Radiation Sciences Program, to design and implement a continuing education course for primary maternity care providers, the first of its kind in Ontario. The core curriculum team of five midwifery and sonography faculty members met monthly for over a year to develop the course and the curriculum. Two Maternal Fetal Medicine specialists also met with the core team as needed to assist in defining course learning objectives as well as to review and review module content.

The course objectives and POCUS indications, such as gestational dating and viability, were informed by a needs assessment which examined the intended uses of ultrasound among Ontario midwives. The curriculum was also guided by the obstetrical competency requirements from Sonography Canada Sonography Canada Guidelines and Member Policies 2019 ( Ling, 2019). The format for the course, which combined online learning, hands-on practice and continued learning under supervision in practice, was based on two existing continuing education courses – emergency skills in obstetrics and a point of care ultrasound in the emergency room. Overall, the course encompassed five online modules, a two-day hands-on workshop, and a clinical practicum where learners work toward developing competency of skills. The practicum was guided by a detailed competency logbook that outlined specific skills across trimesters. The learners worked directly with a qualified sonography preceptor who oversaw their achievement of competencies.

Online module topics included: sonography ethics, the physics of ultrasound, and sonographic protocols throughout pregnancy. The modular curriculum was primarily created by the sonography faculty, who drew on their obstetrics content and learning objectives from their existing medical radiation sciences program. Content related to how sonography competencies apply to the midwifery scope of practice, including discussions of the scope, ethics, and limitations of ultrasound were created by the midwifery faculty and the maternal fetal medicine specialists. For example, if abnormal findings or urgent presentations such as bleeding were discovered using POCUS, a diagnostic scan referral was required.

The hands-on, 2-day workshop allowed learners to practice the clinical skills introduced in the modules with computer simulations and pregnant volunteers. Over 40 pregnant volunteers across all stages of pregnancy were recruited through midwifery clinics in Hamilton Ontario via social media and clinic flyers to provide simulated learning that would closely represent their clients. To support learners during the workshop, the learner to instructor ratio was 3:1 so learners could receive lots of real-time feedback on their scans and opportunities to ask questions. Elements that were taught and the competencies that midwives gained through this course included: intrauterine pregnancy, crown-rump length, and mean sac diameter in the first trimester. For trimester two/three, indications included fetal heart rate, biparietal diameter, head circumference, abdominal circumference, femur length, and placentation.

After successful completion of the workshop, learners were expected to organize a practicum in their home community to further develop their skills under the guidance of a preceptor.

Learner knowledge, skills, and attitudes were continuously assessed across the 3 modalities of instruction. Participants were required to complete the modules (approximately twenty hours to complete) and attain a minimum of 80% on formative quizzes before attending the workshop. Ultrasound skills were evaluated during the workshop through a series of simulated scans as part of an objective structural clinical exam (OSCE). These respective assessments were developed by the sonography faculty based on other medical radiation sciences OSCE content. Learners were required to pass the OSCE to progress to the practicum and remediation was available if required. In the practicum, learners maintained a logbook of completed first, second and third trimester scans, all of which were evaluated by a sonography preceptor. Logbook criteria were developed and implemented by sonography faculty based on the learning objectives, and strategies to confirm proficiency. Learners were required to complete 20 first and second/third trimester real-time scans respectively. During the practicum, learners worked directly with their preceptor to seek clarification and guidance, ensuring they were well-supported in advancing their POCUS skills.

 Successful completion of all course materials and assessments entitled learners a certificate of course completion. Additional details of the course content and educational methods are in Table 1.

**Table 1.** POCUS Course Curriculum.

Course Content	Learning Method	Assessment Method
Ultrasound Physics	Online Module	Module Quiz
Sonography Ethics	Online Module	Module Quiz
Sonography Scanning Techniques	Two Day Workshop Clinical Practicum	OSCE Logbook Assessment
Anatomy Scans	Online Module Two Day Workshop Clinical Practicum	Module Quiz OSCE Logbook Assessment
**First Trimester**		
Intrauterine Pregnancy	Module Two Day Workshop Clinical Practicum	Module Quiz OSCE Logbook Assessment
Establishing Expected Delivery Date	Module	Module Quiz
Identifying Singleton Pregnancy	Module	Module Quiz
Assessing Early Pregnancy Viability	Two Day Workshop Clinical Practicum	OSCE Logbook Assessment
**Second Trimester**		
Confirming Pregnancy Viability	Two Day Workshop Clinical Practicum	OSCE Logbook Assessment
**Third Trimester**		
Fetal Presentation	Module Two Day Workshop Clinical Practicum	Module Quiz OSCE Logbook Assessment
Placental Location	Module Two Day Workshop Clinical Practicum	Module Quiz OSCE Logbook Assessment
Amniotic Fluid	Module Two Day Workshop Clinical Practicum	Module Quiz OSCE Logbook Assessment

Seventeen midwives enrolled and completed the POCUS course. Learners’ course evaluation encouraged reflection on the course structure and content. Survey data from the learners indicated that they appreciated the diversity of topics and educational methods used. The learners shared how the course gave them a well-rounded understanding of the knowledge and clinical skills required to incorporate POCUS throughout antenatal care. Participants indicated they would use bedside scanning most frequently to assess viability and confirmation of fetal presentation. Learner feedback highlighted how they appreciated the diversity of topics and lots of opportunity to practice scanning in the workshop. Certain aspects of the course were challenging for learners; for example, the physics module was cited as being difficult due to the depth and volume of content. It was also noted that the course and the gaining of new ultrasound skills took more time than anticipated. Finally, due to challenges with the coronavirus disease 2019 pandemic, with limited availability of clinical sites to take on additional learners, many struggled to complete the clinical practicum; however by 2024, half of learners had completed the practicum.

## Discussion

The interprofessional collaboration during the curriculum development and implementation of this course ensured that the content was strongly rooted in pregnancy-specific sonography competencies. The course was a feasible and effective approach for promoting knowledge, skills, and judgment for qualified midwives to add POCUS to their clinical scope of practice. Strengths of this curriculum included the use of various teaching and learning modalities that encompassed didactic and clinical skills teachings and a clinical practicum guided by competency-based assessments.

Reflecting on the first cohort, the inclusion of theoretical knowledge and practical skills was very important to ensure that providers could successfully and appropriately perform a POCUS scan. Although some leaners commented on the material being difficult, this content needs to be continued, potentially over a longer time-period as sonography is a complex, operator-dependent modality. The challenges midwives experienced in coordinating their clinical practicum, is a continued theme across clinical education programs where placement availability is limited. Specifically, organizing placements has been shown to be difficult because the increased number of learners and limited resources available in clinical settings, which was worsened over the pandemic ( Larue *et al*., 2015). Potentially in this POCUS course, instructors could provide more time on the ultrasound simulator and with pregnant volunteers in the workshop to reduce the number of scans required in the placement or sonography preceptors providing skills instruction in a midwifery clinic. Considerations of POCUS in gynecology care may be an area for course expansion. Areas of strength include incorporating multiple educational methods that encompassed didactic and experiential approaches to thoroughly introduce sonography. Learners’ positive reception to the course re-iterates midwives’ interest in using POCUS to aid their clinical decisions ( Johnston *et al*., 2023; Johnston *et al*., 2024). As this was the first iteration of this POCUS course, it is not possible to comment on the long-term impacts of this training. However, future research aimed at understanding how learners have continued to apply their POCUS training in practice would be valuable.

## Conclusions and future directions

The education offered in the McMaster-Mohawk POCUS course was well received by midwives. Based on the success of the first cohort, the course has continued to run and is now an elective in the Master’s of Midwifery Program at McMaster University. There have been four subsequent cohorts to complete the course and some course refinements based on learner feedback have been made. Specifically, logbook assessments and case requirements have been modified to improve flexibility in obtaining on-going clinical competence. The two-day workshop has continued with pregnant volunteers across gestational ages. Future research should address how learners continue to maintain and apply POCUS over extended time.

In sharing our experiences with designing and implementing this POCUS course, we hope to provide guidance for curricula developers to help make POCUS more widely available in clinical care.

## Data Availability

No data are associated with this article.
