# Supplementary material for: Insights into an innovative point of care ultrasound curriculum for Ontario primary maternity care providers
Source: MedEdPublish (2016). 2025 Feb 24;12:64. Originally published 2022 Oct 26. [Version 3] doi: 10.12688/mep.19361.3 (PMC11871427; doi:10.12688/mep.19361.3)
Supplement: Peer review history

# Reviewer report: Reviewer response for version 3

**DOI:** 10.21956/mep.22430.r41116

**Published:** 2025-02-28

**Authors:** Carolynne J Cormack

**Affiliations:** 1 Monash University, Clayton, Victoria, Australia

Thank you for the opportunity to re-review this revised paper (version 3). My compliments to the authors for addressing all peer review feedback. I note in particular the addition of a curricular content table and details which significantly enhance the paper.

Is the case presented with sufficient detail to be useful for teaching or other practitioners?

Partly

Is the work clearly and accurately presented and does it cite the current literature?

Yes

If applicable, is the statistical analysis and its interpretation appropriate?

Not applicable

Is the case’s background and context in sufficient detail?

Yes

Are all the source data underlying the results available to ensure full reproducibility?

No

Are the conclusions drawn adequately supported by the results?

Yes

Reviewer Expertise:

Sonographer with specific expertise in POCUS education, curricular development and health professional education research.

I confirm that I have read this submission and believe that I have an appropriate level of expertise to confirm that it is of an acceptable scientific standard.

# Reviewer report: Reviewer response for version 2

**DOI:** 10.21956/mep.22099.r39119

**Published:** 2024-10-17

**Authors:** Johannes Weimer

**Affiliations:** 1 University Mainz, Mainz, Germany

Thank you for the opportunity to review this manuscript. The manuscript provides and comprehensive insight into the development of a POCUS curriculum, offering information for other educational institutions interested in creating similar programs. However, the manuscript would benefit from more detailed data on course outcomes and challenges encountered during its implementation. Additionally, a stronger focus on long-term impacts and evaluation methods for the program’s success would enhance the depth and practical relevance of the manuscript. I have added further details and suggestions for improvement:

**Abstract**

 The abstract provides a clear overview of the topic and the goals of the manuscript. It outlines the development of the POCUS program and how it was integrated into practice. Key points, such as interdisciplinary collaboration and the benefits of the program, are well communicated.

 The abstract could be more precise by including specific results and challenges faced during the implementation. For instance, how was the program's success measured? the OSCE results should be implemented. The programme was developed 5 years ago, has no further course been run since then?

**Introduction**

 The introduction effectively explains the relevance of POCUS in clinical midwifery practice. It clearly outlines how ultrasound is integrated into professional practice and why it was necessary to develop a dedicated course.

 The introduction could benefit from additional statistical data on POCUS usage to support the need for this program. The transition between describing the problem and introducing the course solution could be smoother. Furthermore, a more detailed explanation of existing gaps in midwifery education would help underscore the necessity of this curriculum.

**Results**

 The results section provides a good description of the course development process, the involvement of various disciplines, and the structured curriculum design. The description of the course components, including online modules and hands-on training, is clear and comprehensive.

 More quantitative data would strengthen this section, such as the number of hours or sessions dedicated to each module or practicum. Additionally, more details on how learners were supported and evaluated during the hands-on training would better demonstrate the program’s practical value.

 Including more statistical data, such as the percentage of participants who completed the modules and practicum successfully, would enhance the results. Additionally, a more detailed analysis of participant feedback would be beneficial, highlighting which aspects of the course were particularly well-received or required improvement. The OSCE results should be implemented.

**Discussion**

 The discussion effectively reflects on the interdisciplinary collaboration and highlights the strengths and weaknesses of the program. It offers practical suggestions for future iterations of the course and potential expansions.

 This section could benefit from a deeper exploration of how the challenges (e.g., lack of practicum sites) could be addressed in future versions of the program. Furthermore, discussing the long-term impact of the program on midwifery practice would be valuable. The inclusion of a follow-up system to measure long-term learning outcomes would also be a beneficial addition.

Is the case presented with sufficient detail to be useful for teaching or other practitioners?

Partly

Is the work clearly and accurately presented and does it cite the current literature?

Partly

If applicable, is the statistical analysis and its interpretation appropriate?

Partly

Is the case’s background and context in sufficient detail?

Partly

Are all the source data underlying the results available to ensure full reproducibility?

Partly

Are the conclusions drawn adequately supported by the results?

Yes

Reviewer Expertise:

I am currently working as a researcher in a medical didactic institute. My research expertise is in the development and implementation of ultrasound-specific educational formats. This includes the development of training concepts, curricula and examination formats.The methodological focus is on blended learning or flipped classroom in the context of prospective studies (observational studies, randomized studies, proof-of-concept studies).The future aspect of digitization, simulator training and the use of artificial intelligence in medical education is also a focus.I am currently familiar with the necessary statistical calculations and their interpretation.

I confirm that I have read this submission and believe that I have an appropriate level of expertise to confirm that it is of an acceptable scientific standard, however I have significant reservations, as outlined above.

# Reviewer report: Reviewer response for version 2

**DOI:** 10.21956/mep.22099.r39120

**Published:** 2024-10-06

**Authors:** Carolynne J Cormack

**Affiliations:** 1 Monash University, Clayton, Victoria, Australia

Thank you for the opportunity to review this revised paper. This paper makes a valuable contribution to the growing literature about point of care ultrasound use by midwives in obstetric care. 

**I would make the following comments:**

 1. There has been no inclusion of detail of the curricular content or OSCE criteria. Given that the title is 'insights into an innovative POCUS curriculum' I strongly suggest inclusion of a table or other form of supplemental data to briefly outline the curriculum. What elements of first and second/third trimester sonography were taught and what competencies were achieved by midwives in this case study? 

 eg: even main headings of what was covered in teaching content and assessment would be informative

 Ultrasound physics & concepts

 Equipment & image acquisition (scan presets, depth, gain etc) 

 Scan technique, protocols & measurements 

 T1 - confirmation of IUP, dating (CRL, MSD).

 T2 - FHR, biometry (BPD, HC, AC, FL), placentation.

 T3 - presentation, AFI, biometry (BPD, HC, AC, FL), wellbeing.

 I understand word limits constrain detail, however this is very important information for other ultrasound educators who may wish to attempt to replicate a similar teaching program. Including this as supplemental data does not impact word limits.

 2. Keywords - suggest the authors use recognised MeSH search terms 

 3. There are some minor grammatical errors that need correction. 

 Abstract - 'many benefits into learning', suggest rewording

 Abstract - use of term 'Fall' is specific term to US/Canada, suggest only refer to year 2019

 p4. 'simulation scans an' should be 'in an'

 p4. 'their existing other medical', suggest rewording

Is the case presented with sufficient detail to be useful for teaching or other practitioners?

Partly

Is the work clearly and accurately presented and does it cite the current literature?

Yes

If applicable, is the statistical analysis and its interpretation appropriate?

Not applicable

Is the case’s background and context in sufficient detail?

Yes

Are all the source data underlying the results available to ensure full reproducibility?

No

Are the conclusions drawn adequately supported by the results?

Yes

Reviewer Expertise:

Sonographer with specific expertise in POCUS education, curricular development and health professional education research.

I confirm that I have read this submission and believe that I have an appropriate level of expertise to confirm that it is of an acceptable scientific standard, however I have significant reservations, as outlined above.

# Reviewer report: Reviewer response for version 1

**DOI:** 10.21956/mep.20747.r34354

**Published:** 2024-07-15

**Authors:** Phuc Nhon Nguyen

**Affiliations:** 1 Tu Du Hospital, Ho Chi Minh City, Vietnam

Congratulations for your work. Although this entity is not a new insight, I read with interest POCUS at the authors center. This assessment is considered as important primary care in the improvement of women’s health as well as in other specialities.

 In reality, not only the midwives and nurses, the obstetricians also can use the sonography machine placed in the delivery room in order to monitor the labor stage, fetal head position and station. 

 Interestingly, POCUS is also used during labor induction such as Foley, Cook insertion and examination of the fetal presentation after integration. A lot of cases are diagnosed to return to breech presentation (altered fetal presentation) in the condition of small for gestational age fetus and polyhydramnios. Maslovitz et al. have reported 14 out of 1083 cases of pregnant women who underwent transcervical Foley for induction of labor (Maslovitz S, Lessing JB, Many A. Complications of trans-cervical Foley catheter for labor induction among 1,083 women. Arch Gynecol Obstet. 2010 Mar;281(3):473-7. doi: 10.1007/s00404-009-1136-7. Epub 2009 Jun 2. PMID: 19488776.)

 Ultrasound also has a role in predicting the successful induction of labor:

 This must be great use of POCUS at the bedside. Thus, I have some suggestions to improve the worthy paper:

- The Introduction is too short. It must be expanded more, including how the clinical course happened before application of POCUS.
- Clinical indication related to obstetric issues given with POCUS (bedside assessment of pregnancy dating and fetal presentation) should be added. For example, determining the fetal heart position for Doppler auscultation or the abdominal cavity fluid in uterine rupture.
- POCUS is used as the simple ultrasound, thus the midwives could use it to monitor the patient. But POCUS is used after an accurate ultrasound scan followed by an experienced sonographer or whenever. The authors should describe how the protocol is used if they detect an abnormal result during ultrasound (abnormal placental implantation including placenta previa, placenta accreta spectrum disorders) or the problems associated with amniotic fluid volume (oligohydramnios).
- I wonder if POCUS is also applied for gynecologic issues such as abnormal uterine bleeding. In case of myoma or endometrial pathology, the endometrial thickness is measured.
  - Nguyen PN, Nguyen VT. Endometrial thickness and uterine artery Doppler parameters as soft markers for prediction of endometrial cancer in postmenopausal bleeding women: a cross-sectional study at tertiary referral hospitals from Vietnam. Obstet Gynecol Sci. 2022 Sep;65(5):430-440. doi: 10.5468/ogs.22053. Epub 2022 Apr 29. PMID: 35488358; PMCID: PMC9483670.
- POCUS can be applied during surgical intervention in the gynecologic field. 
- The authors should describe the result before and after intervention of POCUS at the author’s center.
- The conditions requiring application of POCUS should be mentioned (ultrasound machine, level of hospital, experiences of performers, training, certification). I thought that POCUS is difficult to apply in low-resource settings with a non-trained midwife.
- The authors can add a review literature table that includes all the recent reports in the past 5 years.
- Addition of the real images should have been followed-up during the procedures of POCUS.
- Strengths and limitations should be included.
- A perspective and future implication is necessary for this promising strategy, for example, a RCT study with POCUS application and non-POCUS application. Therefore, this can demonstrate to the world the meaning of POCUS.

Is the case presented with sufficient detail to be useful for teaching or other practitioners?

Yes

Is the work clearly and accurately presented and does it cite the current literature?

Yes

If applicable, is the statistical analysis and its interpretation appropriate?

Not applicable

Is the case’s background and context in sufficient detail?

Partly

Are all the source data underlying the results available to ensure full reproducibility?

Yes

Are the conclusions drawn adequately supported by the results?

Yes

Reviewer Expertise:

Obstetrics and gynaecology

I confirm that I have read this submission and believe that I have an appropriate level of expertise to confirm that it is of an acceptable scientific standard, however I have significant reservations, as outlined above.

## References

[1] : Complications of trans-cervical Foley catheter for labor induction among 1,083 women. *Arch Gynecol Obstet* .2010;281(3) : 10.1007/s00404-009-1136-7 473-7 10.1007/s00404-009-1136-7 19488776

[2] : Endometrial thickness and uterine artery Doppler parameters as soft markers for prediction of endometrial cancer in postmenopausal bleeding women: a cross-sectional study at tertiary referral hospitals from Vietnam. *Obstet Gynecol Sci* .2022;65(5) : 10.5468/ogs.22053 430-440 10.5468/ogs.22053 35488358 PMC9483670

# Reviewer report: Reviewer response for version 1

**DOI:** 10.21956/mep.20747.r34784

**Published:** 2023-10-12

**Authors:** Ruchika Karnik

**Affiliations:** 1 Yale University, New Haven, CT, USA

I was honored to review the article "Insights into an innovative point of care ultrasound curriculum for Ontario primary maternity care providers".

 The article is concise and well written. It does incorporate innovative educational methods that would be important to disseminate.

 While it provides a great overview of the process, some of the details are missing.

1. The authors alluded to having done a needs assessment, but there is no supplemental material provided to understand what the needs assessment showed and whether that was reflected in the curriculum.
2. How many scans were the learners required to meet as a minimum in their log book to pass the practicum?
3. Learners course evaluation surveys are not included? What was the content of these surveys and did they align well with the needs assessment to show improvement?

In my opinion, it would be crucial to address these questions in order to be able to be reproducible.

Is the case presented with sufficient detail to be useful for teaching or other practitioners?

Partly

Is the work clearly and accurately presented and does it cite the current literature?

Yes

If applicable, is the statistical analysis and its interpretation appropriate?

I cannot comment. A qualified statistician is required.

Is the case’s background and context in sufficient detail?

Yes

Are all the source data underlying the results available to ensure full reproducibility?

No

Are the conclusions drawn adequately supported by the results?

Yes

Reviewer Expertise:

Echocardiography, medical education

I confirm that I have read this submission and believe that I have an appropriate level of expertise to confirm that it is of an acceptable scientific standard, however I have significant reservations, as outlined above.
